# *TREX1* 531C>T Polymorphism is Associated with High Proviral Load Levels in HTLV-1-Infected Persons

**DOI:** 10.3390/v12010007

**Published:** 2019-12-19

**Authors:** Denis de Castro Silva, Ednelza da Silva Graça Amoras, Tuane Carolina Ferreira Moura, Felipe Teixeira Lopes, Samara Tatielle Monteiro Gomes, Carlos A. da Costa, Maísa Silva Sousa, Ricardo Ishak, Antonio Carlos Rosário Vallinoto, Maria Alice Freitas Queiroz

**Affiliations:** 1 Laboratório de Virologia, Instituto de Ciências Biológica, Universidade Federal do Pará, Belém 66.075-110, Brazil; denisdecastrosilva@gmail.com (D.d.C.S.); ednelza@hotmail.com (E.d.S.G.A.); tuanecfmoura@gmail.com (T.C.F.M.); feliptlopes1@gmail.com (F.T.L.); samara_tatielle@yahoo.com.br (S.T.M.G.); rishak@ufpa.br (R.I.); vallinoto@ufpa.br (A.C.R.V.); 2 Laboratory of Cellular and Molecular Biology, Tropical Medicine Center, Federal University of Pará, Belém 66.055-240, Brazil; carauco@gmail.com (C.A.d.C.); maisaufpa@gmail.com (M.S.S.)

**Keywords:** HTLV-1, TREX1, polymorphism, proviral load, ANA, cytokines, HAM/TSP

## Abstract

Human T-lymphotropic virus type 1 (HTLV-1) deregulates the immune system and cell cycle, resulting in loss of immune tolerance and disease, including HTLV-1-associated myelopathy/tropical spastic paraparesis (HAM/TSP). Three prime repair exonuclease 1 (TREX1) maintains innate immune tolerance of the host and host-cell permissiveness to retroviral infections. TREX1 polymorphisms may influence the course of infection and autoimmune manifestations. The influence of TREX1 531C/T polymorphism was investigated in HTLV-1 infection and development of symptoms among 151 persons infected with HTLV-1 (32 HAM/TSP, 19 rheumatologic manifestations, two dermatitis, five more than one diagnosis, two probable HAM/TSP, and 91 asymptomatic individuals) and 100 uninfected persons in the control group. Polymorphism genotyping and proviral load quantification were performed by real-time polymerase chain reaction (PCR) and antinuclear antibodies (ANAs) were screened by an indirect immunofluorescence assay. No statistically significant difference was found in polymorphism genotype and allele frequencies between the infected and control groups. HAM/TSP patients showed higher frequency of TT genotype than asymptomatic persons (*p* = 0.0339). Proviral load was significantly higher among individuals with CT/TT genotypes and CC genotype carriers had lower proviral load and higher levels of proinflammatory cytokines. ANAs were present only in the HAM/TSP group. TREX1 531C>T polymorphism seems to be associated with TREX-1 regulation and HTLV-1 infection.

## 1. Introduction

Human T-lymphotropic virus type 1 (HTLV-1) is a retrovirus responsible for the development of different types of diseases, including adult T-cell leukemia/lymphoma and HTLV-1-associated myelopathy/tropical spastic paraparesis (HAM/TSP), in addition to other inflammatory syndromes [1,2,3,4]. Virus infection is endemic in certain regions such as Japan, Australia, Melanesia, the Caribbean, the Middle East, and South America, and it is estimated that 5 to 10 million people are infected worldwide [5].

The pathogenesis of HAM/TSP is a result of the interaction between the virus and the host immune system, including innate and adaptive immunity mechanisms [6]. Innate immunity is important for host defense against viral infections as it regulates viral replication, mainly through innate effector factors [7]. Restriction factors inhibit replication, inducing the activation of type I interferon (IFN) production, whereas permissiveness factors may facilitate or promote retrovirus replication [8,9].

TREX-1 is able to recognize and degrade dsDNA and ssDNA molecules present in cytosol, contributing to innate immune tolerance of the host and inhibiting activation of autoimmune mechanisms [10,11]. On the other hand, TREX-1 renders cells permissive to retroviral infections inhibiting the activation of the innate immune response, mainly related to type I IFN synthesis [9]. Intermediate fragments of the genetic material of retroviruses, which originate from reverse transcription in the cytosol, can be degraded by TREX-1 [11]. Higher TREX-1 activity reduces viral DNA recognition by restriction sensors that promote activation of the IFN-α- and IFN-β-mediated antiviral response against the virus; consequently, greater viral replication will occur [9].

Polymorphisms in the *TREX1* gene are associated with functional alterations of the encoded protein, and are directly related to the development of inflammatory autoimmune diseases and the susceptibility to and progression of HIV-1-related diseases [12,13].

Among the polymorphisms studied, rs11797 (*TREX1* 531C/T) has been investigated in the development of autoimmune diseases [12,14,15,16]. However, to date, there is no information on the association of the polymorphism with HTLV-1 infection. The present study investigated the association of the *TREX1* 531C>T polymorphism with the susceptibility to HTLV-1 infection, the development of infection-related symptoms, and the presence of ANAs.

## 2. Materials and Methods

### 2.1. Study Population

The present study included blood samples from 151 individuals infected with HTLV-1 (32 clinically diagnosed with HAM/TSP, 19 with rheumatologic manifestations, two with dermatitis, five with more than one diagnosis, two with probable HAM/TSP, and 91 asymptomatic), who were treated at the outpatient clinic of the Tropical Medicine Center of the Federal University of Pará. The patients were of both sexes, older than 18 years of age, and not treated with glucocorticoids.

A control group included samples from 100 sex workers of both sexes, exposed to the risk of infection but not infected with HTLV-1/2, HIV-1, hepatitis B or C viruses, *Chlamydia trachomatis*, or syphilis; these samples were used to compare the frequencies of polymorphisms. In order to avoid statistical bias due to population stratification, the control group included individuals residing in the city of Belém, State of Pará, who presented a similar ethnic background to that of the HTLV-1-infected group.

### 2.2. Sample Collection and Storage

A sample containing 10 mL of blood was collected by intravenous puncture using a vacuum collection system containing ethylenediaminetetraacetic acid as an anticoagulant. Samples were centrifuged and separated into plasma and a leukocyte mass. The leukocyte samples were used to extract genomic DNA for analysis of TREX1 531C/T polymorphism and to quantify proviral load. Plasma samples were used for the detection of ANAs. Samples were stored in a freezer at −70 °C until use.

### 2.3. DNA Extraction

DNA was extracted from peripheral blood leukocytes using the Puregene kit (Gentra Systems, Minneapolis, MN, USA) according to the manufacturer’s protocol, which included cell lysis, protein precipitation, and DNA precipitation and rehydration. DNA was quantified using a Qubit® 2.0 fluorometer (Life Technologies, Carlsbad, CA, USA) and Qubit™ DNA assay kit reagents (Life Technologies, Carlsbad, CA, USA), following the protocol recommended by the manufacturer.

### 2.4. Quantification of HTLV-1 Proviral Load

Proviral load was quantified using a quantitative real-time PCR using three target sequences, synthesized through the TaqMan® system (Life Technologies, Foster City, CA, USA), according to a previously described protocol [17]. Samples containing 5 mL of whole blood were collected for leukocyte DNA extraction, followed by relative quantification using real-time PCR. The results were subsequently adjusted for the absolute proviral quantity, based on leukocyte counts per mm^3^, and expressed as proviral DNA copies/mm^3^.

### 2.5. Genotyping of TREX1 531C/T (rs11797)

TREX1 531C/T polymorphism, located in exon 16 of the gene, was analyzed by real-time PCR using a StepOnePLUS™ real-time PCR system (Thermo Fisher, Carlsbad, CA, USA). The reaction used a commercial assay (C11537906-20) containing primers and probes specific for the amplification of the target sequence. The reaction contained 1× master mix, H_2_O, 20× assay C_11537906_20, and 50 ng of DNA. The PCR cycling conditions were as follows: 10 min at 95 °C and 40 cycles of 15 s at 95 °C and 1 min at 60 °C.

### 2.6. ANA Screening in HEp-2 Cells

The detection of autoantibodies against cellular antigens was performed by indirect immunofluorescence, a technique that identifies antibodies which bind to their substrate antigens, using anti-immunoglobulin G (anti-IgG) molecules conjugated to fluorescein, according to V Brazilian Consensus Guidelines for detection of anti-cell autoantibodies in HEp-2 cells [18]. Samples were tested using a commercial substrate, ANA HEp-2 diagnostic test, and the VIRGO® ANA/HEp-2 IgG indirect fluorescent antibody kit (Hemagen Diagnostics, Columbia, MD, USA) according to the manufacturer’s instructions. Slides were prepared using serum in 1:80 dilution. The slides were observed under a Nikon Eclipse E-200 fluorescence microscope (Nikon Instruments Inc., Melville, NY, USA) using a 10× eyepiece lens and a 40× objective lens. We also used a ZOE fluorescent cell imager fluorescence microscope (Bio-Rad, Hercules, CA, USA) to record the fluorescence patterns.

### 2.7. Quantification of Plasma Cytokines

Plasma cytokines (Tumor necrosis factor alpha—TNF-α, interferon Gamma—IFN-γ, and Interleukin 10—IL-10) were measured by the Ready-SET-Go® enzyme-linked immunosorbent assay (ELISA) (eBioscience, San Diego, CA, USA), which uses specific monoclonal antibodies to detect the cytokine. Procedures followed the manufacturer’s instructions.

### 2.8. Statistical Analysis

The genotype and allele frequencies were estimated by direct counting, and the significance of differences between groups was calculated using the χ^2^ (chi-squared) test and the *G*-test. The Hardy–Weinberg equilibrium was calculated to assess whether the distribution of the observed genotype frequencies matched the expected frequencies. Sample sizes of proviral load were small and the statistical analyses were performed using a bootstrap test, that is, a hypothesis *t*-test with 1000 resamples, and the *p*-value nearest to the value of real data was considered. Comparison of cytokine levels was performed using the Mann–Whitney test. All tests were performed using the BioEstat 5.3 software (Belém, PA, Brasil) [19], and *p*-values < 0.05 were considered statistically significant.

### 2.9. Ethics Statement 

The project was approved by the Research Ethics Committee of the Health Sciences Institute of the Federal University of Pará (protocol no. 2872434/2018; 4 September 2018). All study participants were informed about the research objectives, and those who agreed to participate signed an informed consent form.

## 3. Results

The Hardy–Weinberg equilibrium calculation showed that the genotype frequencies found in both groups were as expected (*p* > 0.05). The frequency of the wild-type genotype was high in all groups investigated. No significant differences were observed in the genotypes and alleles between the HTLV-1-infected and the uninfected persons (Table 1). No significant difference was observed when comparing asymptomatic individuals with HTLV-1-infected patients (Table 2); however, patients with HAM/TSP had a higher frequency of the TT genotype than asymptomatic individuals (*p* = 0.0339; Table 3).

**Figure 1:**
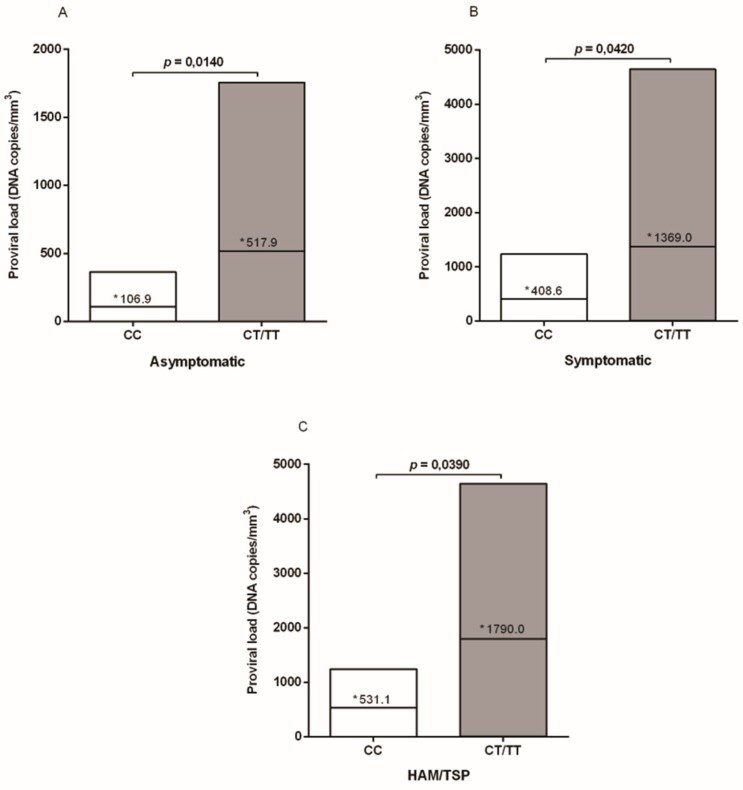
HTLV-1 proviral loads according to the TREX1 531C/T polymorphism genotypes in asymptomatic and symptomatic individuals. * Mean values.

The mean proviral load levels among the individuals with the three genotypes were different in each of the investigated groups (asymptomatic CC: 106.9, CT: 500.5, and TT: 548.2; symptomatic CC: 408.6, CT: 1013, and TT: 1473; HAM/TSP CC: 531.1, CT: 1792, and TT: 2213). However, the analyses were performed comparing individuals with wild genotype with those with at least one polymorphic allele, since it was observed that CT and TT individuals presented higher levels of proviral load, and in order to avoid stratification of the sample number of the analyzed groups. Proviral load was significantly higher among those individuals with the CT/TT genotypes, in the group of asymptomatic persons (*p* = 0.0140; Figure 1A), in patients with symptoms (*p* = 0.0420; Figure 1B), and among those clinically diagnosed with HAM/TSP (*p* = 0.0390; Figure 1C).

**Figure 2:**
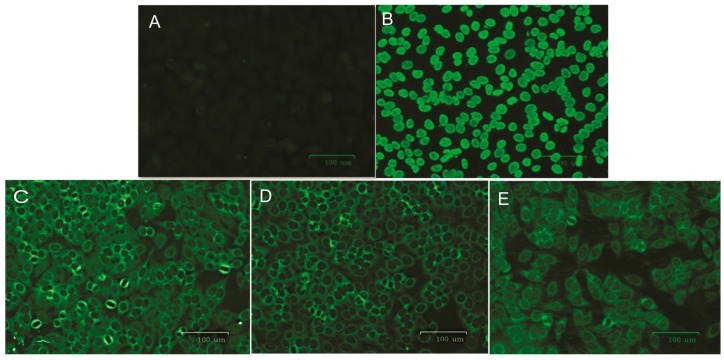
ANAs test patterns. (**A**) Negative control; (**B**) homogeneous nuclear positive control; (**C**) sample 1; (**D**) sample 2; and (**E**) sample 3, which showed a homogeneous cytoplasmic pattern of fluorescence. Serum dilution factor: 1:80.

ANAs were detected among 30% of patients with HAM/TSP but not in the asymptomatic group (Table 4). TREX1 531C/T polymorphism according to the presence of ANAs showed that two of the three ANAs positive patients carried CC genotype (20%) and one presented the CT genotype (10%). All three ANA-positive patients had a homogeneous cytoplasmic pattern of fluorescence (Figure 2C–E).

**Figure 3:**
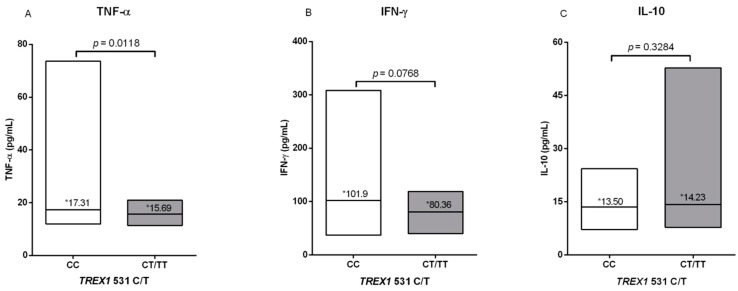
Comparison of (**A**) TNF-α, (**B**) IFN-γ, and (**C**) IL-10 cytokine levels between wild (CC) and polymorphic (CT/TT) genotypes of TREX1 531C/T polymorphism. * Median values.

Evaluation of cytokine levels showed that carriers of the CC allele had higher levels of TNF-α and IFN-γ proinflammatory cytokines, while carriers of the CT/TT polymorphic genotypes had higher levels of IL-10, although only the evaluation of TNF-α was statistically significant (Figure 3).

## 4. Discussion

The efficiency of HTLV-1 transmission between hosts is lower than that of other retroviruses [20,21]. In addition, few infected individuals develop any disease; therefore, the identification of genetic factors of the host that are potentially associated with the susceptibility to HTLV-1-related diseases has been described [22,23].

The present study investigated the possible influence of TREX1 531C>T polymorphism with HTLV-1 infection and the progression to disease. The frequency of the polymorphic allele was 0.25 in the control group and ranged from 0.25 to 0.31 in the infected group. Similar frequencies have been reported in public databases for African (0.27) and Asian (0.31) populations [24]. The allele frequencies of the polymorphism apparently did not differ between different ethnic groups, including an extensive ethnically mixed population, such as the one investigated herein [25]. 

In the present study, the association of TREX1 531C>T polymorphism with susceptibility to HTLV-1 infection was not observed, as previously reported of other polymorphisms in HIV-1 infection [13,26]. However, we did not analyze all possible variables of exposure to infection between the two groups that could allow the evaluation of the role of polymorphism in the susceptibility to HTLV-1 infection.

The investigation of genotype frequencies and the presence of symptoms showed individuals with a higher frequency of TT genotype with HAM/TSP. In addition, HTLV-1 proviral load was higher in both symptomatic and asymptomatic persons carrying polymorphic T alleles (CT/TT genotypes). These results suggest that TREX1 531C>T polymorphism seems to influence TREX-1 regulation and HTLV-1 infection. A possible mechanism could be related to the reduction of degradation of viral dsDNA and ssDNA molecules in the cytosol [10,11] that may contribute to the development of HAM/TSP, since it would reduce the activation of type 1 IFN production which is responsible for antiviral control, thus allowing increased viral replication and, consequently, the development of symptoms.

The increase of proviral load is a consolidated risk factor associated with the development of HAM/TSP and for rheumatoid diseases, such as rheumatoid arthritis and connective tissue diseases [3,27].

Therefore, identification of genetic factors that may influence the evolution of the infection are important for a better understanding of the pathogenesis and the establishment of improved follow-up strategies, allowing the detection of early identification biomarkers on the onset of symptoms and reducing the risk of disease development.

Retroviruses are well-known etiologic agents of autoimmune and rheumatic disorders. The investigation of serum autoimmune markers (such as ANAs, antineutrophil cytoplasmic antibodies, and rheumatoid factors) among HTLV-1-infected persons is a good strategy to assess this association, although a recent study has reported the low frequency of these markers among HTLV-1-infected individuals in Iran [28].

In the present study, three patients with HAM/TSP were positive for ANAs, presenting a cytoplasmic dense fine pattern of fluorescence, but none of the asymptomatic individuals were. ANAs prevalence has been reported in patients with HAM/TSP [29], and a study carried out in Colombia showed that 70.2% of HAM/TSP patients were ANAs positive, compared to 27.27% of HTLV-1-infected asymptomatic persons [30], showing that disease development was accompanied by autoantibodies.

The pattern of cytoplasmic dense fine fluorescence is observed because of antibodies reacting with PL-7, PL-12, and antiribosomal P protein cell antigens. The same pattern is observed in systemic lupus erythematosus, polymyositis, and dermatomyositis [31].

The ANA-positive patients showed reactivity up to a dilution of 1:160. Initial screening for ANAs is important for the diagnosis of autoimmune diseases, and individuals with a positive ANAs test should be further investigated for autoimmune diseases, and given appropriate treatment.

The number of HTLV-1-infected persons in each geographical setting may be low, and this situation compromises the use of appropriate statistical testing. Because of the small sample size, the association of ANAs with TREX1 531C>T polymorphism genotypes limits the strength of conclusions but allows the suggestion of a possible influence of ANAs with the C wild allele in the development of HAM/TSP, since two of the individuals presented the CC genotype and one the CT genotype. However, these results are not in agreement with the information presenting the same polymorphism in relation to systemic lupus erythematosus in which the T allele was associated with the disease [15]. In the present study, the presence of ANAs may not be directly related to the wild genotype, probably due to the small sample size of HTLV-1-infected individuals. Thus, further studies with a larger sampling are important to better define the role of TREX1 531C>T variation genotypes in ANAs production and the development of HAM/TSP, and whether other factors may also induce the production of these autoantibodies. Development of HAM/TSP is associated with autoimmunity, either by the mechanism above or by the equivocal triggering of an auto-reaction to cell antigens similar to HTLV-1 antigens and the resulting inflammatory process [32].

The role of TREX1 531C>T polymorphism in the development of autoimmune diseases is not yet well understood. Although it is not associated with some autoimmune diseases [14], a higher prevalence of this variation was observed in patients with systemic lupus erythematosus and Sjögren’s syndrome [15,16]. 

The possible role of TREX1 531C>T polymorphism has not yet been investigated in relation to the infection or progression of infectious diseases. Considering the unique characteristics of the genetics of the population investigated that result from interethnic crossings of Europeans, Indians, and Africans [25], this led us to the suggestion that the polymorphism (or possibly its haplotype in linkage disequilibrium) [12] may somehow influence TREX-1 regulation and HTLV-1 infection, contributing to increased proviral load and HAM/TSP pathogenesis in the tri-hybrid population investigated. 

Additionally, even though this polymorphism was not associated with the presence of antinuclear antibodies, it seems to contribute, along with other factors, to increased proviral load and HAM/TSP pathogenesis in the population evaluated.

INF type I induces the establishment of an antiviral state in the infected cells and also towards neighboring cells, and modulates innate immunity and activation of the adaptative immune response [33]. The levels of cytokines involved with T helper 1 (Th1) and Th2 immune responses in the present investigation showed that individuals with CC wild genotype presented higher levels of pro-inflammatory cytokines (TNF-α, IFN-γ) and lower proviral load, while those with polymorphic genotypes (CT/TT) showed higher levels of IL-10 and higher proviral load. This information suggests a possible occurrence of TREX alterations, induction in the production of IFN type I, and activation of an effective TH1 immune response against HTLV-1 infection. The results observed may suggest a possible association among the investigated immunological variables in relation to HTLV-1 infection; however, we do understand that the results need further investigations to confirm the role of TREX1 and the exact mechanisms involved.

In summary, the TREX1 531C>T polymorphism was associated with HAM/TSP, which seems to alter the regulation of TREX-1, favoring HTLV-1 replication and higher proviral load. However, polymorphism was not related to the presence of autoantibodies, which appear to be influenced by other factors independent of polymorphism.

## Figures and Tables

**Table 1:** Genotype and allele frequencies of three prime repair exonuclease 1 (TREX1) 531C/T polymorphism among human T-lymphotropic virus type 1 (HTLV-1) carriers and in the control group.

Genotypes and Alleles	HTLV-1 *n* = 151 n (%)	Control *n* = 100 n (%)	*p* *
CC	83 (54.97)	60 (60.00)	0.6095
CT	56 (37.09)	31 (31.00)	
TT	12 (7.94)	9 (9.00)	
C	0.74	0.75	0.7243
T	0.26	0.25	

n, number of individuals. * Chi-squared test.

**Table 2:** Genotype and allele frequencies of TREX1 531C/T polymorphism among asymptomatic and symptomatic HTLV-1 carriers.

Genotypes and Alleles	Asymptomatic *n* = 91 n (%)	Symptomatic *n* = 58 n (%)	*p*
CC	48 (52.74)	32 (57.63)	0.2151 #
CT	38 (41.76)	18 (30.51)	
TT	5 (5.50)	8 (11.86)	
C	0.74	0.72	0.8735 *
T	0.26	0.28	

n, number of individuals. * Chi-squared test. # *G*-test.

**Table 3:** Genotype and allele frequencies of TREX1 531C/T polymorphism among asymptomatic HTLV-1 carriers and patients with HTLV-1-associated myelopathy/tropical spastic paraparesis (HAM/TSP) and rheumatologic manifestations.

Genotypes and Alleles	Asymptomatic *n* = 91 n (%)	HAM/TSP *n* = 32 n (%)	Rheumatologic Manifestations *n* = 19 n (%)	*p*1	*p*2
CC	48 (52.74)	19 (59.38)	8 (42.10)	0.0339 #	0.6241 #
CT	38 (41.76)	7 (21.87)	9 (47.37)		
TT	5 (5.50)	6 (18.75)	2 (10.53)		
C	0.74	0.75	0.69	1.000 *	0.5309 *
T	0.26	0.25	0.31		

n, number of individuals. * Chi-squared test. # *G*-test. *p*1, asymptomatic vs. HAM/TSP; *p*2, asymptomatic vs. rheumatologic manifestations.

**Table 4:** Prevalence of antinuclear antibodies (ANAs) among selected individuals from the asymptomatic and HAM/TSP groups.

Genotypes and Alleles	Asymptomatic *n* = 10 n (%)	HAM/TSP n = 10 *n* (%)
ANA POS	0 (0.0)	3 (30.0)
ANA NEG	10 (100.0)	7 (70.0)

n, number of individuals.
